# Controlled human malaria infection in Kenyan adults: A safe model that could accelerate assessment of novel drugs and vaccines in malaria endemic populations

**DOI:** 10.1186/1475-2875-13-S1-O33

**Published:** 2014-09-22

**Authors:** Susanne Hodgson, Elizabeth Juma, Amina Salim, Charles Magiri, Domtila Kimani, Daniel Njenga, Alfred Muia, Andrew Cole, Caroline Ogwang, Peter Billingsley, Eric James, Kim Lee Sim, Thomas Rampling, Adrian Hill, Faith Osier, Simon Draper, Philip Bejon, Stephen Hoffman, Bernhards Ogutu, Kevin Marsh

**Affiliations:** 1 The Jenner Institute, University of Oxford, Oxford, UK; 2 Kenyan Medical Research Institute, Nairobi, Kenya; 3 Kenyan Medical Research Insitute, Kilifi, Kenya; 4 Sanaria, Rockville, Maryland, USA

## Background

Controlled human malaria infection (CHMI) studies, where healthy volunteers are infected with *Plasmodium falciparum* have become a vital tool to accelerate vaccine and drug development. As CHMI trials are carried out in a controlled environment, they allow unprecedented, detailed evaluation of parasite growth dynamics and immunological responses to infection. Though commonly performed in malaria-naïve populations, CHMI trials have rarely been performed in malaria endemic regions, despite the advantages of early testing of candidate anti-malaria vaccines and drugs in populations where malaria is endemic. To date, modern CHMI studies have not been used to investigate mechanisms of naturally acquired immunity (NAI) to *P. falciparum* infection.

## Materials and methods

We conducted an open label, randomized CHMI pilot study using aseptic, cryopreserved *P. falciparum* sporozoites (PfSPZ Challenge) administered intramuscularly to evaluate safety, infectivity and parasite growth dynamics in healthy Kenyan adults (n = 28). We included adults with varying degrees of prior exposure to malaria in order to allow an evaluation of the effect of NAI on parasite growth dynamics post CHMI.

## Results

All participants developed blood-stage infection, with a similar safety profile to that observed in northern CHMI centres. One volunteer remained asymptomatic and blood-film negative until day 21 post injection of PfSPZ Challenge, despite developing confirmed blood-stage infection by quantitative polymerase chain reaction (qPCR). This volunteer had a notably reduced parasite multiplication rate (1.3) in comparison to the other 27 volunteers (median 11.1). A significant correlation was seen between parasite multiplication rate (PMR) and anti-schizont ELISA OD at screening (*P* = 0.044).

## Conclusions

Our study has shown that the PfSPZ Challenge is safe and infectious in malaria endemic populations and could be used to aid early assessment of the efficacy of candidate malaria vaccines and drugs in African populations. Whilst our findings are limited by sample size, our pilot study proves that NAI can impact on PMR post-CHMI in a detectable fashion. Our findings suggest the CHMI sporozoite model could allow identification of a heterogeneous group of participants with varying degrees of NAI for whom the relationship between PMR and candidate assays of blood-stage immunity can be examined to try to gain greater understanding of the mechanisms of NAI to *P. falciparum* malaria.

